# Supplementary material for: Evaluation of Phytotoxic and Cytotoxic Effects of Prenylated Phenol Derivatives on Tomato Plants (Solanum lycopersicum L.) and Botrytis cinerea B-05 Spores
Source: Plants (Basel). 2025 Oct 27;14(21):3277. doi: 10.3390/plants14213277 (PMC12608347; doi:10.3390/plants14213277)
Supplement: Supplementary file 1 [file plants-14-03277-s001.zip › plants-3908834-supplementary.pdf]

Article

Evaluation of Phytotoxic and Cytotoxic Effects of Prenylated Phenol Derivatives on Tomato Plants (*Solanum lycopersicum* L.) and *Botrytis cinerea* B-05 Spores

Gerard Núñez [1], Ligia Llovera [1], Dioni Arrieche [1], Romanet Berrios [1], Mauricio Soto [1], Mauricio Osorio-Olivares [2], Andrés F. Olea [2], Efraín Sarmiento [2], Azucena González [3], Héctor Carrasco [2,*] and Lautaro Taborga [1,*]

[1] Departamento de Química, Universidad Técnica Federico Santa María, Valparaíso 2340000, Chile; gerard.nunez@sansano.usm.cl (G.N.); ligia.llovera@usm.cl (L.L.); dioni.arrieche@sansano.usm.cl (D.A.); romanet.berrios@usm.cl (R.B.); mauricio.sotoc@usm.cl (M.S.)
[2] Grupo QBAB, Instituto de Ciencias Aplicadas, Facultad de Ingeniería, Universidad Autónoma de Chile, Av. del Valle Sur 534, Santiago 8580640, Chile; osomauricio@gmail.com (M.O.-O.); andres.olea@uautonoma.cl (A.F.O.); efrain.sarmiento@cloud.uautonoma.cl (E.S.)
[3] Instituto de Ciencias Agrarias, Consejo Superior de Investigaciones Científicas, 28006 Madrid, Spain; azu@ica.csic.es
* Correspondence: hector.carrasco@uautonoma.cl (H.C.); lautaro.taborga@usm.cl (L.T.)

Abstract

The phytopathogenic fungus *Botrytis cinerea*, which causes gray mold disease, has become a limiting factor on agricultural production. *B. cinere*a field control is made mainly using chemical fungicides, which has led to the spreading of resistant populations of this fungus. Thus, the quest of new fungicides molecules has been focused on synthesis of natural product-inspired compounds. The main aim of this work is to synthesize prenylated phenol derivatives and to assess their potential application as antifungal agents with minimal phytotoxic effects. Thus, new prenylphenols (**4**, **5**, and **7**) have been obtained by microwave irradiation with yields ranging from 2.4% to 42.9%, whereas compounds **8** and **9** were synthesized with yields of 25.6% and 54.1%, respectively. The effect of different concentrations of these compounds on *B. cinerea* spore germination, and their phytotoxic effect on tomato (*Solanum lycopersicum* L.) seed germination and root growth, were evaluated. Obtained results indicate that biological activities of all tested compounds are concentration-dependent. Interestingly, compound **7** exhibits the highest antifungal activity against *B. cinerea* spores (IC_{50} < 50 μg/mL) with minimal phytotoxicity on tomato seed germination and root growth. In contrast, compounds **2** and **3** are active against spores (IC_{50} = 461 and 325 μg/mL, respectively) but, at the same time, their phytotoxicity is important at the highest concentrations. These results indicated that the presence of hydroxyl and methyl substituents on the aromatic ring of these compounds induces variations in biological activities, and compound **7** could be a promising candidate as a sporicidal agent.

Keywords: *B. cinerea*; mycelial growth; cytotoxicity; allylphenols; prenylphenols; seed germination; root growth

Supplementary Materials: The following supporting information can be downloaded at: www.mdpi.com/xxx/s1:

- **Figure S1**: 1D-NMR spectrum of 5 using $CDCl_3$ as deuterated solvent
- **Figure S2**: Main heteronuclear correlations 2D HMBC at $^2J_{CH}$ (red) and $^3J_{CH}$ (blue) observed for compound **5**.
- **Figure S3**: selective 1D *NOESY* correlation, signal was irradiated at δ_H 3.30 ppm.
- **Figure S4**: Cytotoxicity assay on spores of *B. cinerea* (B-05) for compound 5. Each assay was carried out in quadruplicate at five different concentrations: 800, 400, 200, 100 and 50 µg/mL, respectively. 1% DMSO was used as a negative control.
- **Figure S5:** Tomato seed germination assay for compound **6** for 7 days. Each treatment was carried in quadruplicate at three different concentrations: 0.20, 0.10 and 0.050 mg/mL, respectively.
- **Figure S6**: Tomato root growth assay for compound **3** measured at 168 h. Root lengths were evaluated as a function of concentration: (A) 0.20 mg/mL; (B) 0.10 mg/mL and (C) 0.050 mg/mL. (For the 5.0 mg/mL concentration, no root growth was observed). EtOH was used as a negative control.
- **Figure S7:** 1D-NMR spectrum of 2-metil-5-(3-metilbut-2-en-1-il)benceno-1,4-diol (**4**).
- **Figure S8:** 2D-NMR spectra of 2-metil-5-(3-metilbut-2-en-1-il)benceno-1,4-diol (**4**).
- **Figure S9:** mass spectrum of 2-metil-5-(3-metilbut-2-en-1-il)benceno-1,4-diol (**4**).
- **Figure S10:** 1D-NMR spectrum of 4-methyl-5-(3-methylbut-2-en-1yl)benzene-1,2-diol (**7**).
- **Figure S11:** 2D-NMR spectra of 4-methyl-5-(3-methylbut-2-en-1yl)benzene-1,2-diol (**7**).
- **Figure S12:** mass spectrum of 4-methyl-5-(3-methylbut-2-en-1yl)benzene-1,2-diol (**7**).
- **Figure S13:** 1D-NMR spectrum of 4-(3-hydroxy-3-methylbutyl)-5-methylbenzene-1,2-diol (**8**).
- **Figure S14:** 2D-NMR spectra of 4-(3-hydroxy-3-methylbutyl)-5-methylbenzene-1,2-diol (**8**).
- **Figure S15:** mass spectrum of 4-(3-hydroxy-3-methylbutyl)-5-methylbenzene-1,2-diol (**8**).
- **Figure S16:** 1D-NMR spectrum of 4-(3-hydroxy-3-methylbutyl)benzene-1,2-diol (**9**).
- **Figure S17:** 2D-NMR spectra of 4-(3-hydroxy-3-methylbutyl)benzene-1,2-diol (**9**).
- **Figure S18:** mass spectrum of 4-(3-hydroxy-3-methylbutyl)benzene-1,2-diol (**9**).

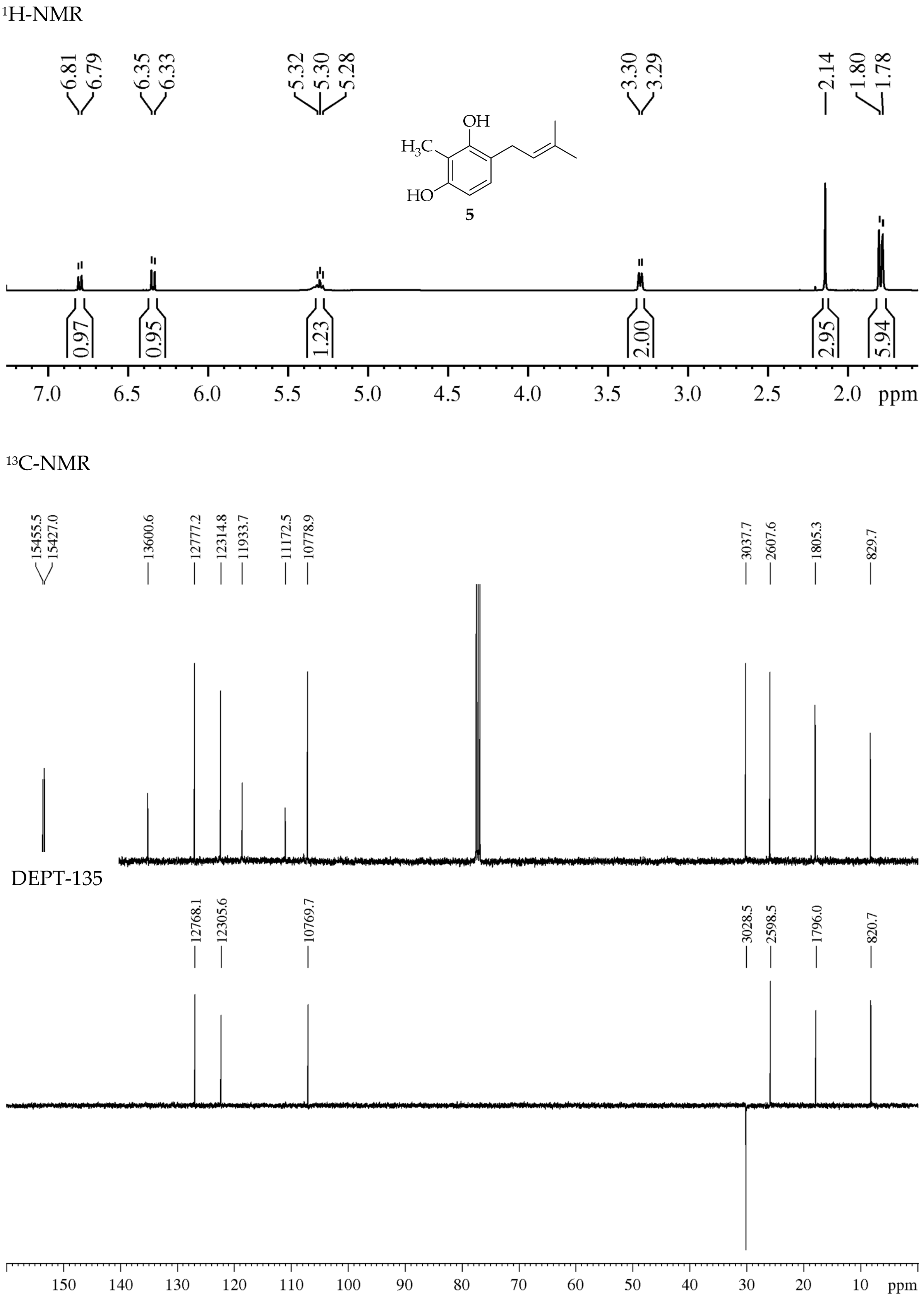

Figure S1: 1D-NMR spectrum of **5** using $CDCl_3$ as deuterated solvent.

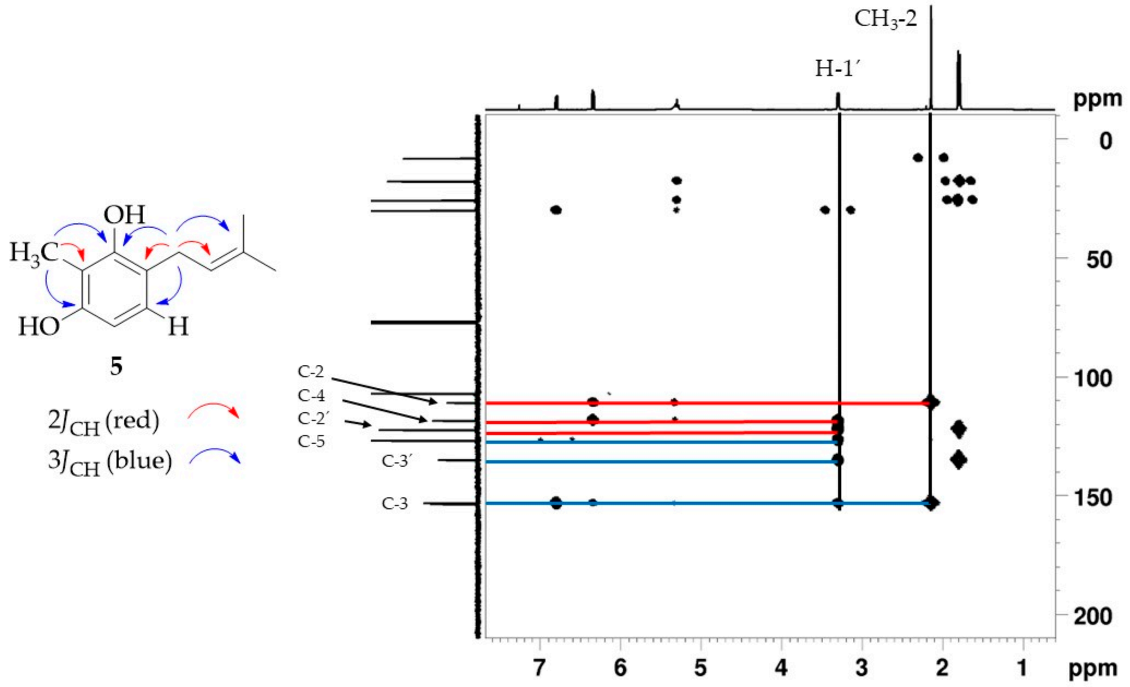

Figure S2: main heteronuclear correlations 2D HMBC at $^{2}J_{CH}$ (red) and $^{3}J_{CH}$ (blue) observed for compound **5**.

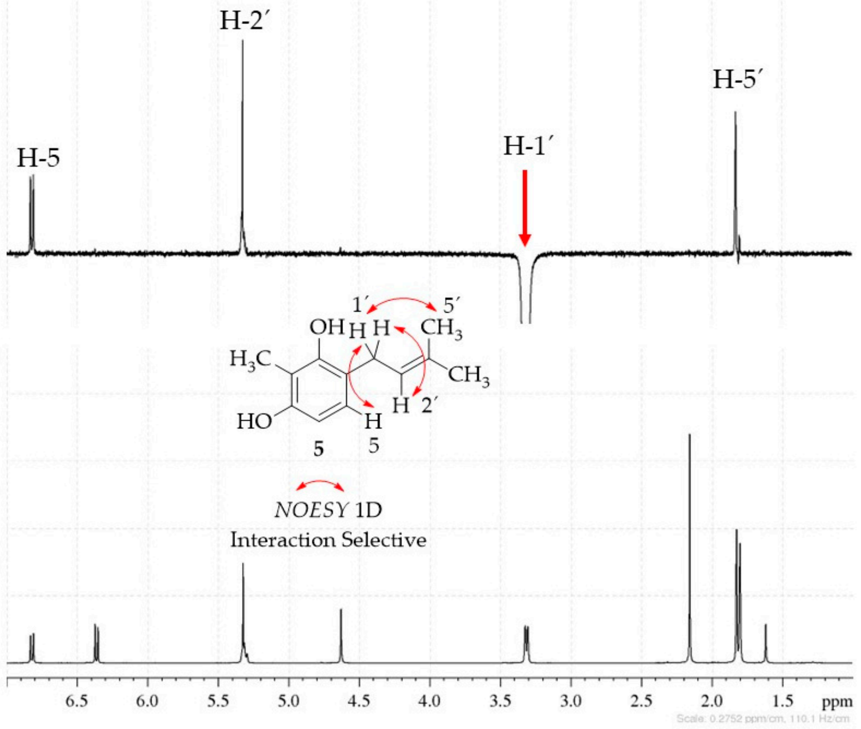

Figure S3: selective 1D *NOESY* correlation, signal was irradiated at δ_H 3.30 ppm**.**

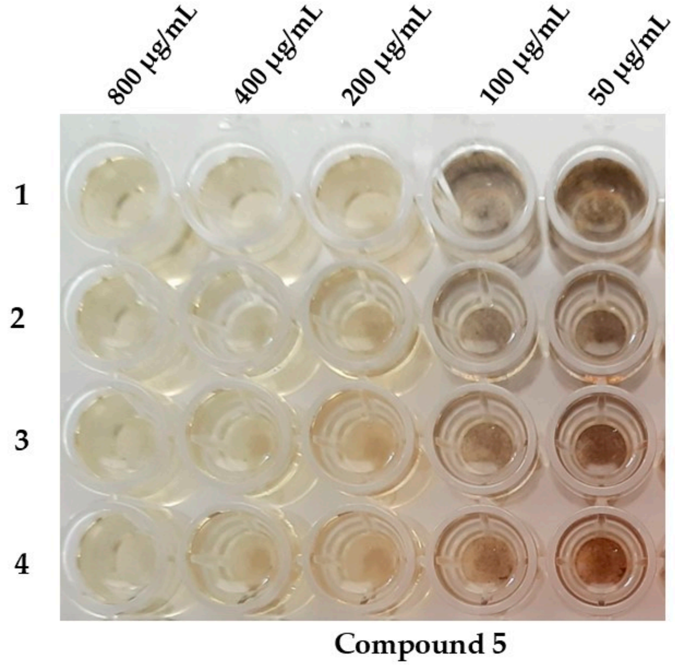

Figure S4: Cytotoxicity assay on spores of *B. cinerea* (B-05) for compound **5**. Each assay was carried out in quadruplicate at five different concentrations: 800, 400, 200, 100 and 50 µg/mL, respectively. 1% DMSO was used as a negative control.

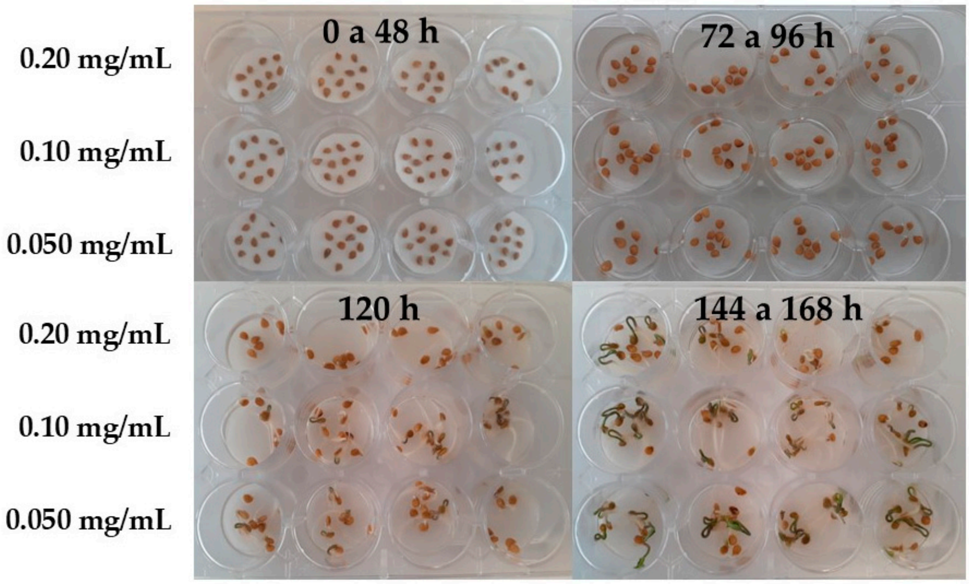

Figure S5: Tomato seed germination assay for compound **6** for 7 days. Each treatment was carried in quadruplicate at three different concentrations: 0.20, 0.10 and 0.050 mg/mL, respectively.

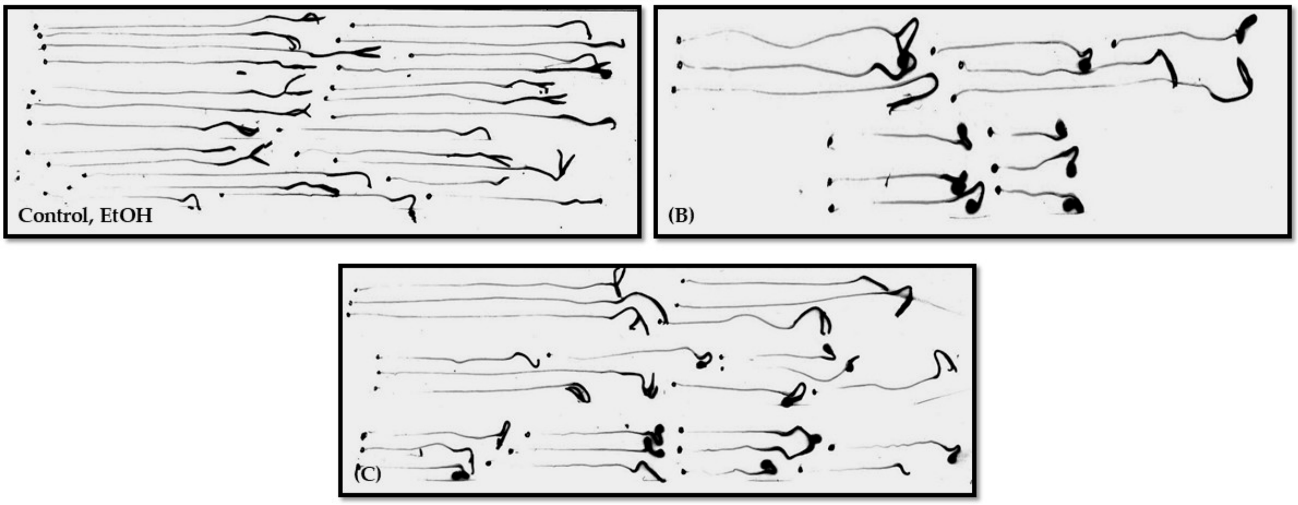

Figure S6: Tomato root growth assay for compound **3** measured at 168 h. Root lengths were evaluated as a function of concentration: (A) 0.20 mg/mL; (B) 0.10 mg/mL and (C) 0.050 mg/mL. (For the 5.0 mg/mL concentration, no root growth was observed). EtOH was used as a negative control.

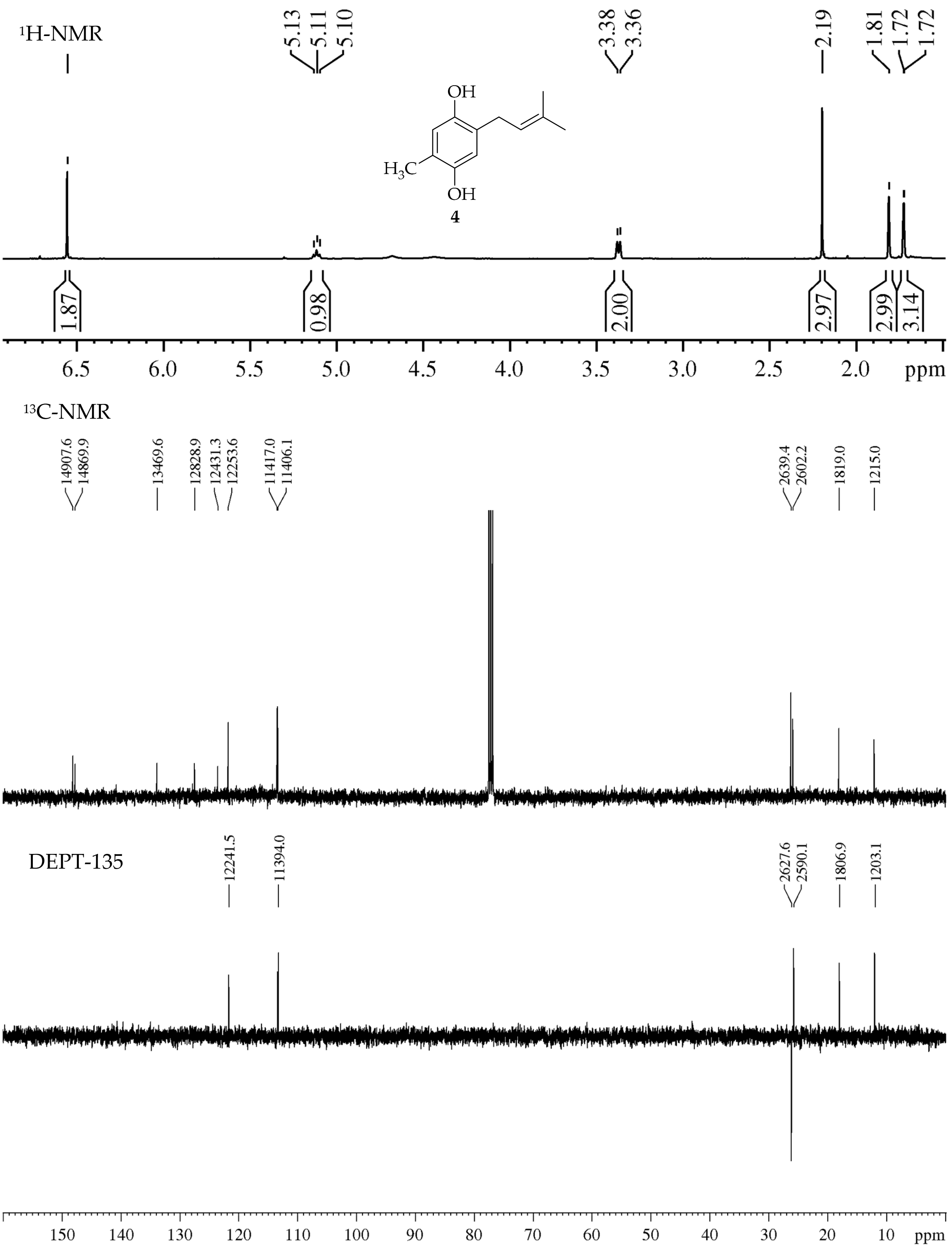

Figure S7: 1D-NMR spectrum of 2-metil-5-(3-metilbut-2-en-1-il)benceno-1,4-diol (**4**).

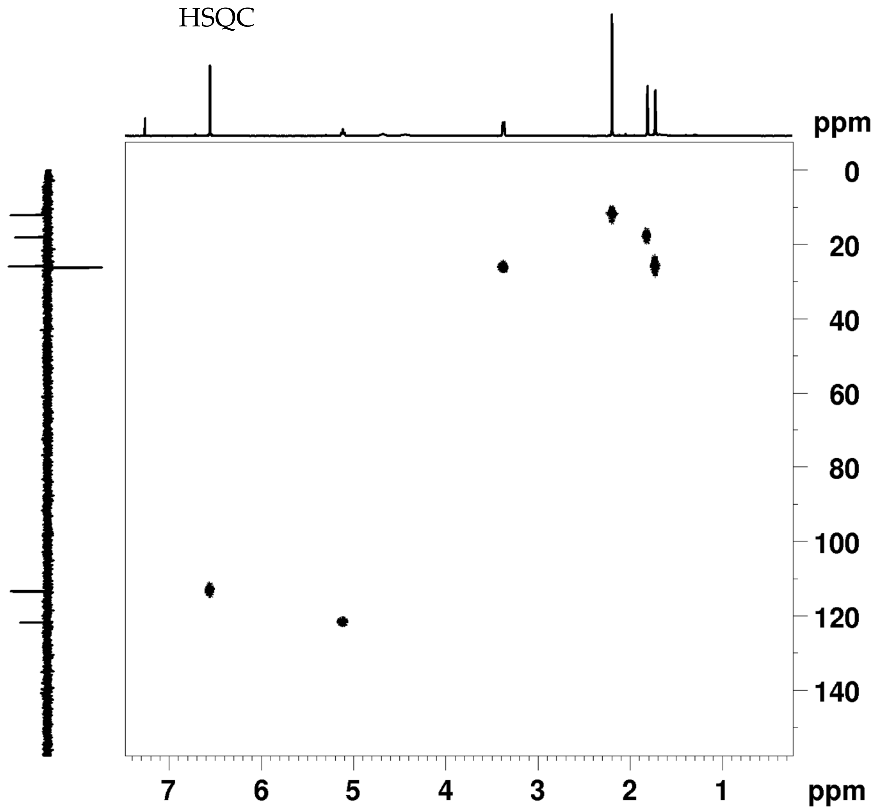

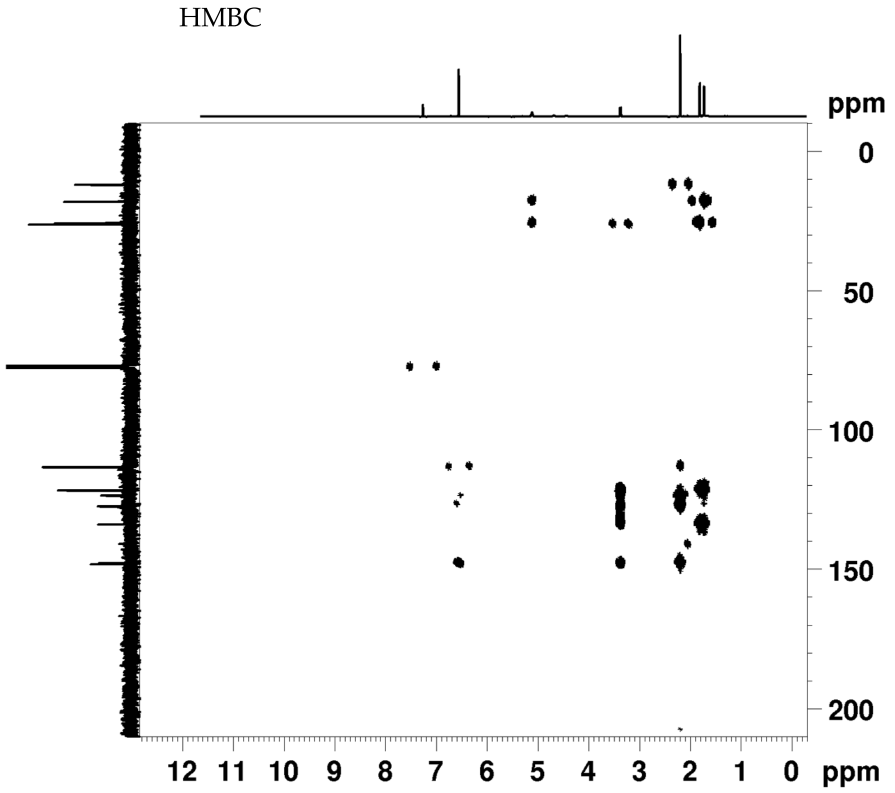

Figure S8: 2D-NMR spectra of 2-metil-5-(3-metilbut-2-en-1-il)benceno-1,4-diol (**4**).

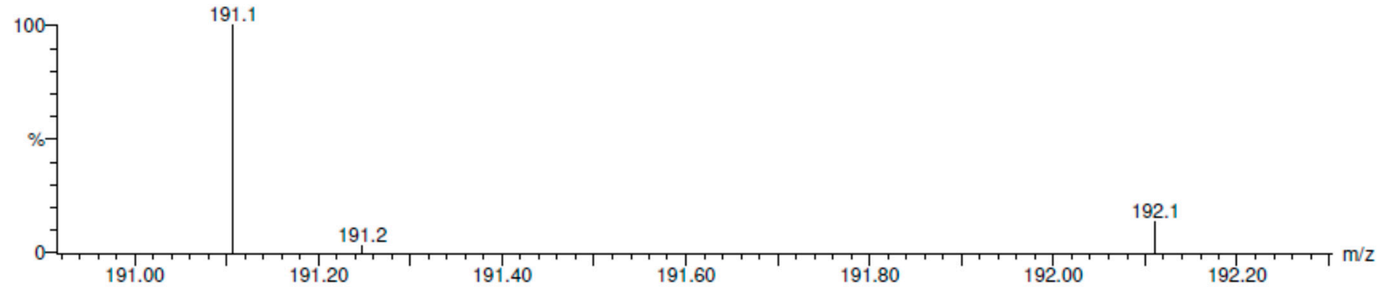

	Mass	RA	Calc. Mass	mDa	PPM	DBE	i-FIT	i-FIT (Norm)	Formula
Minimum:		30.00				-1.5			
Maximum:		100.00		5.0	5.0	100.0			
	191.1072	100.00	191.1072	0.0	0.0	5.5	36.4	0.0	C12 H15 O2

Figure S9: mass spectrum of 2-metil-5-(3-metilbut-2-en-1-il)benceno-1,4-diol (**4**).

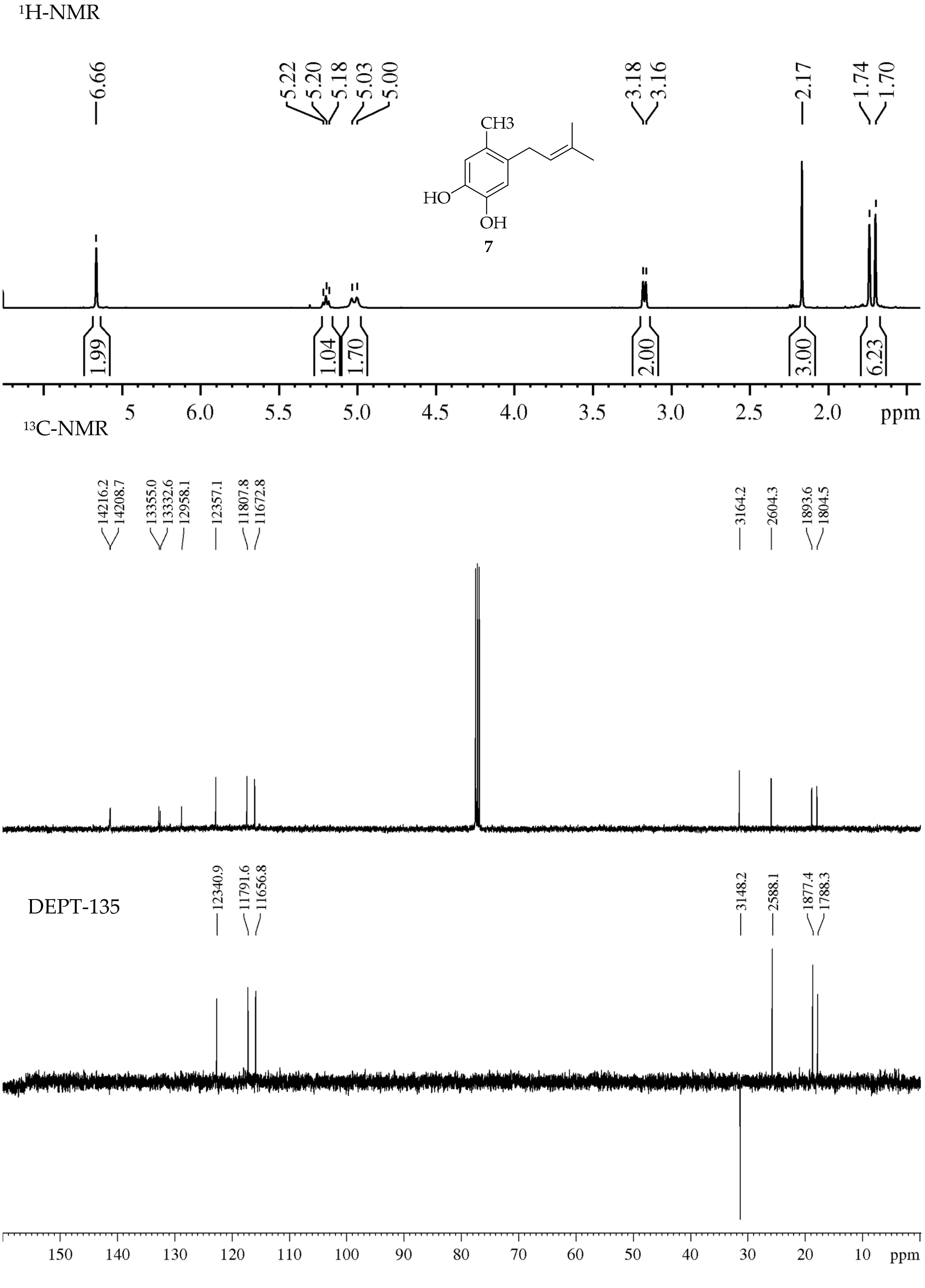

Figure S10: 1D-NMR spectrum of 4-methyl-5-(3-methylbut-2-en-1yl)benzene-1,2-diol (**7**).

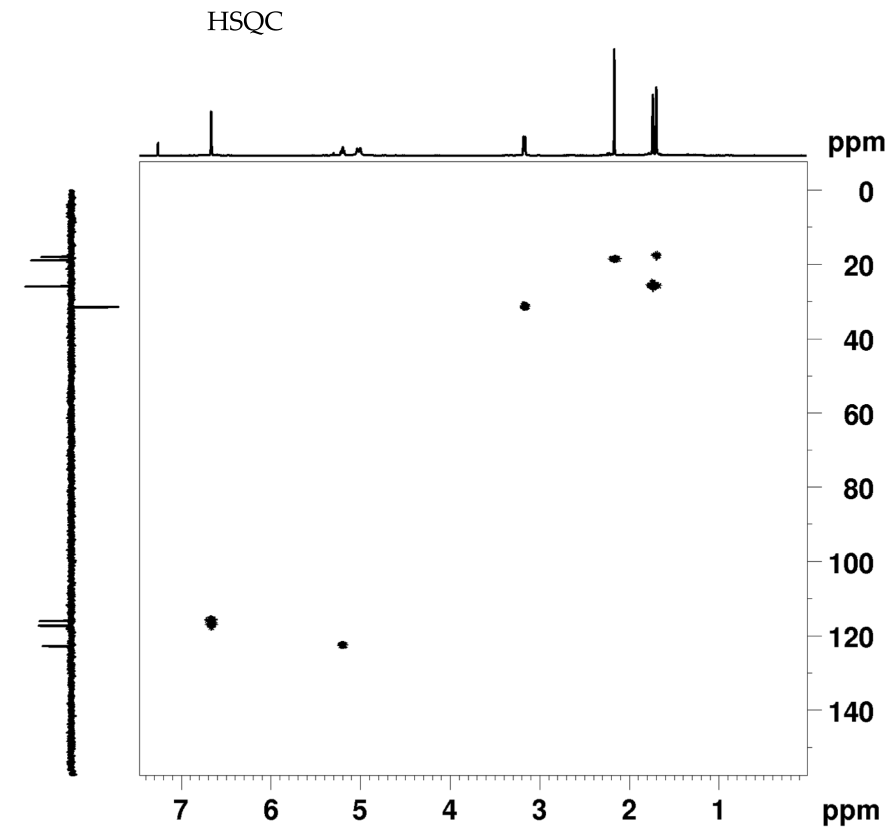

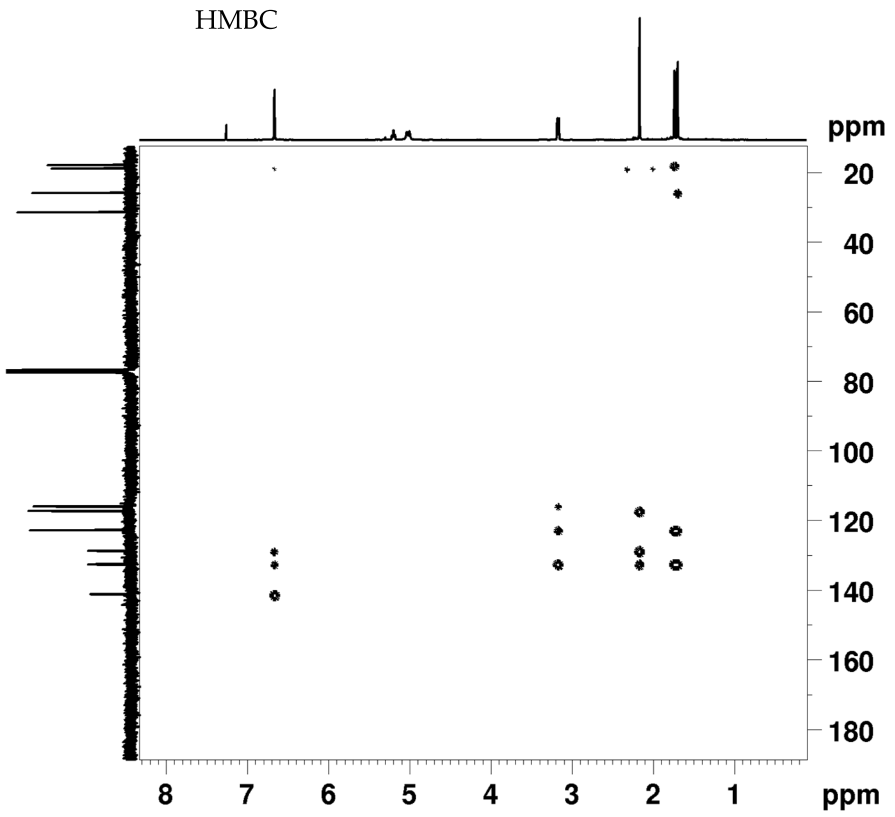

Figure S11: 2D-NMR spectra of 4-methyl-5-(3-methylbut-2-en-1yl)benzene-1,2-diol (**7**).

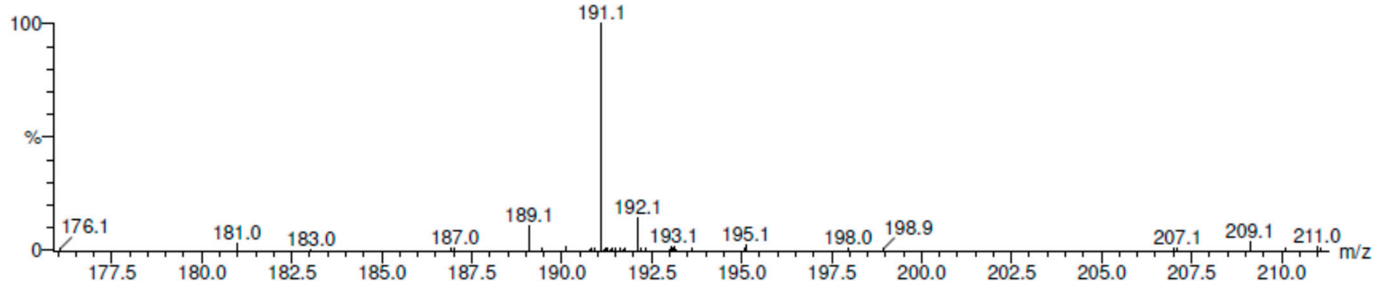

Minimum:	30.00				-1.5			
Maximum:	100.00		5.0	5.0	100.0			
Mass	RA	Calc. Mass	mDa	PPM	DBE	i-FIT	i-FIT (Norm)	Formula
191.1078	100.00	191.1072	0.6	3.1	5.5	76.4	0.0	C12 H15 O2

Figure S12: mass spectrum of 4-methyl-5-(3-methylbut-2-en-1yl)benzene-1,2-diol (**7**).

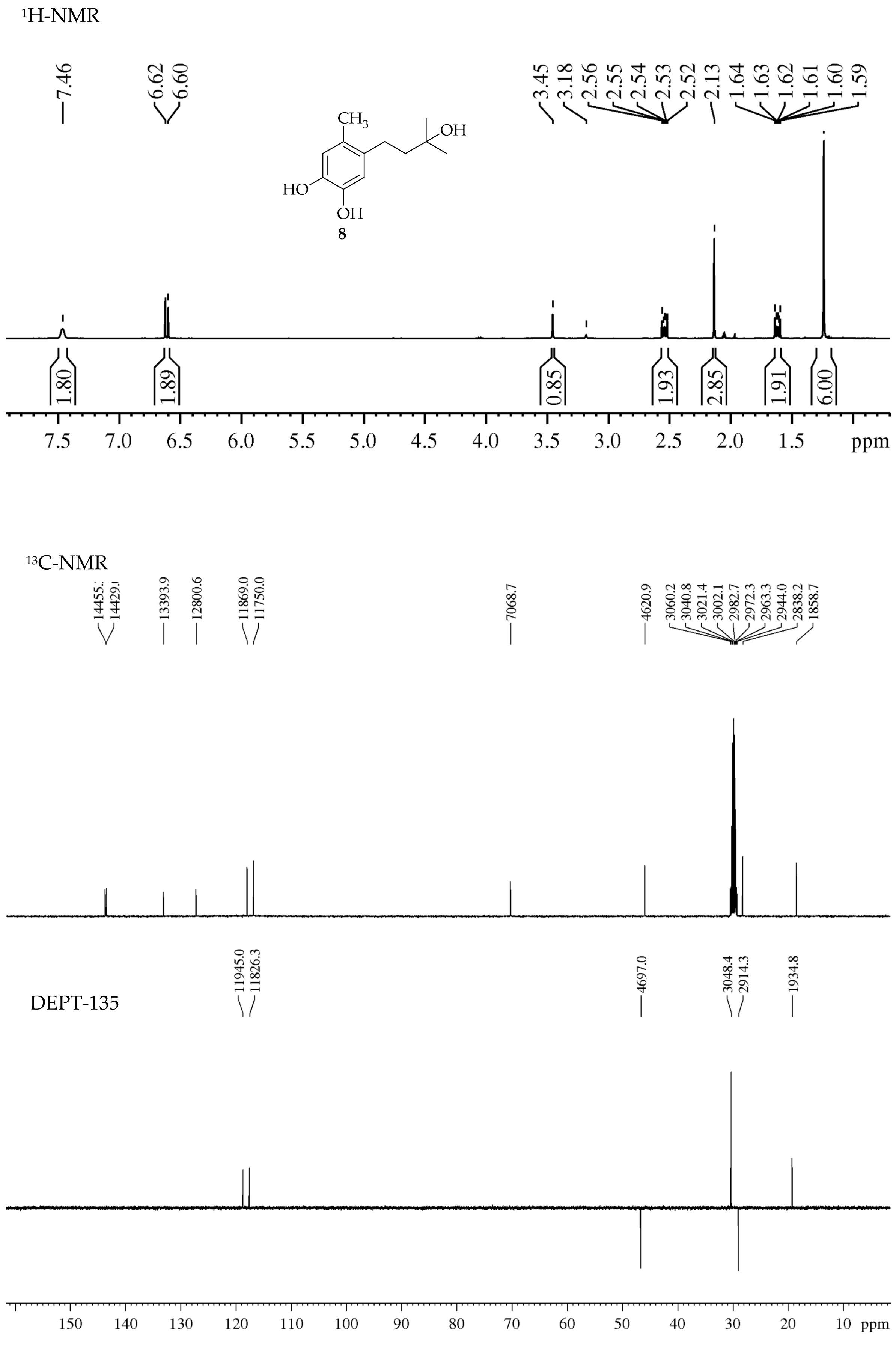

Figure S13: 1D-NMR spectrum of 4-(3-hydroxy-3-methylbutyl)-5-methylbenzene-1,2-diol (**8**).

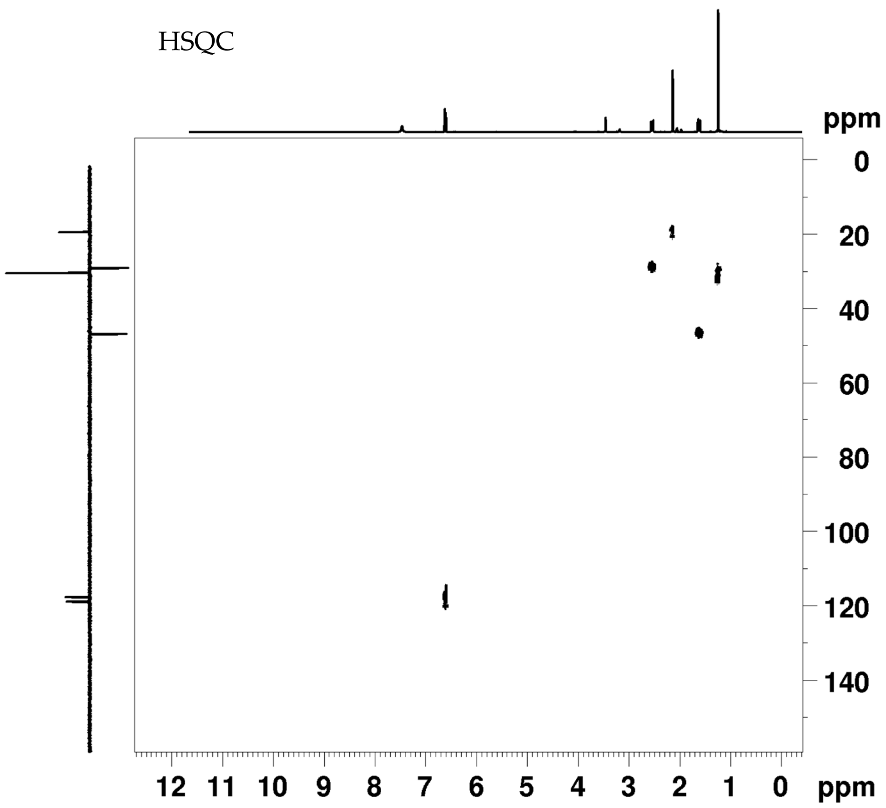

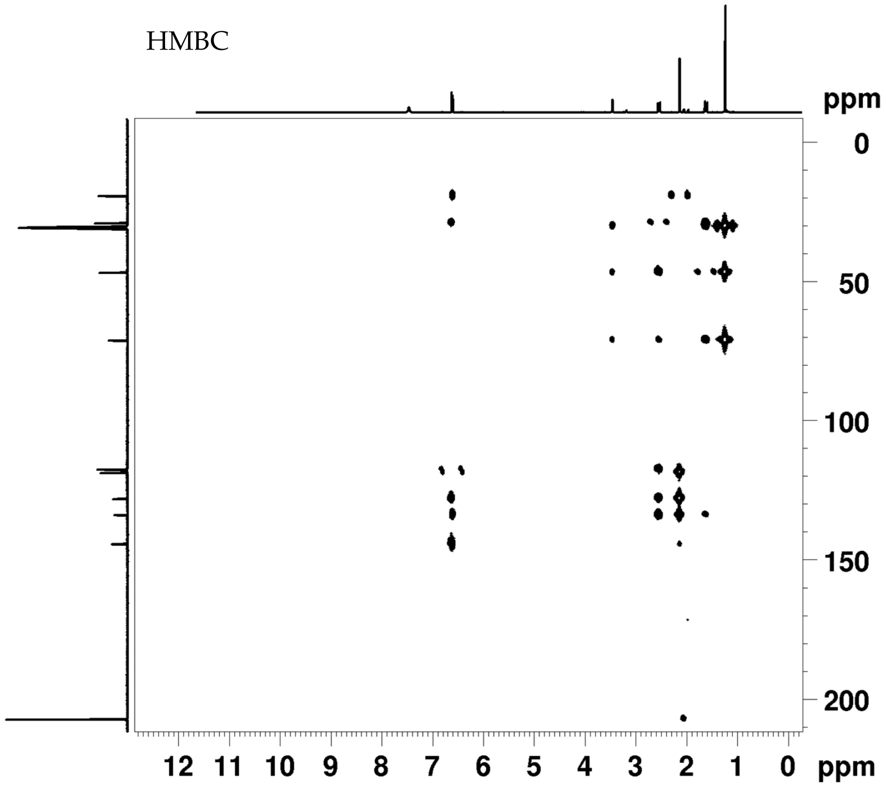

Figure S14: 2D-NMR spectra of 4-(3-hydroxy-3-methylbutyl)-5-methylbenzene-1,2-diol (**8**).

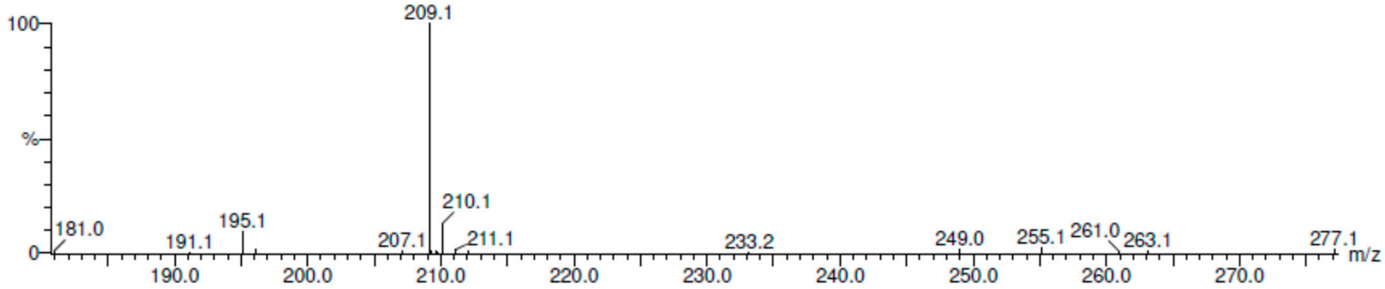

Minimum:	30.00				-1.5			
Maximum:	100.00		5.0	5.0	100.0			
Mass	RA	Calc. Mass	mDa	PPM	DBE	i-FIT	i-FIT (Norm)	Formula
209.1180	100.00	209.1178	0.2	1.0	4.5	32.2	0.0	C12 H17 O3

Figure S15: mass spectrum of 4-(3-hydroxy-3-methylbutyl)-5-methylbenzene-1,2-diol (**8**).

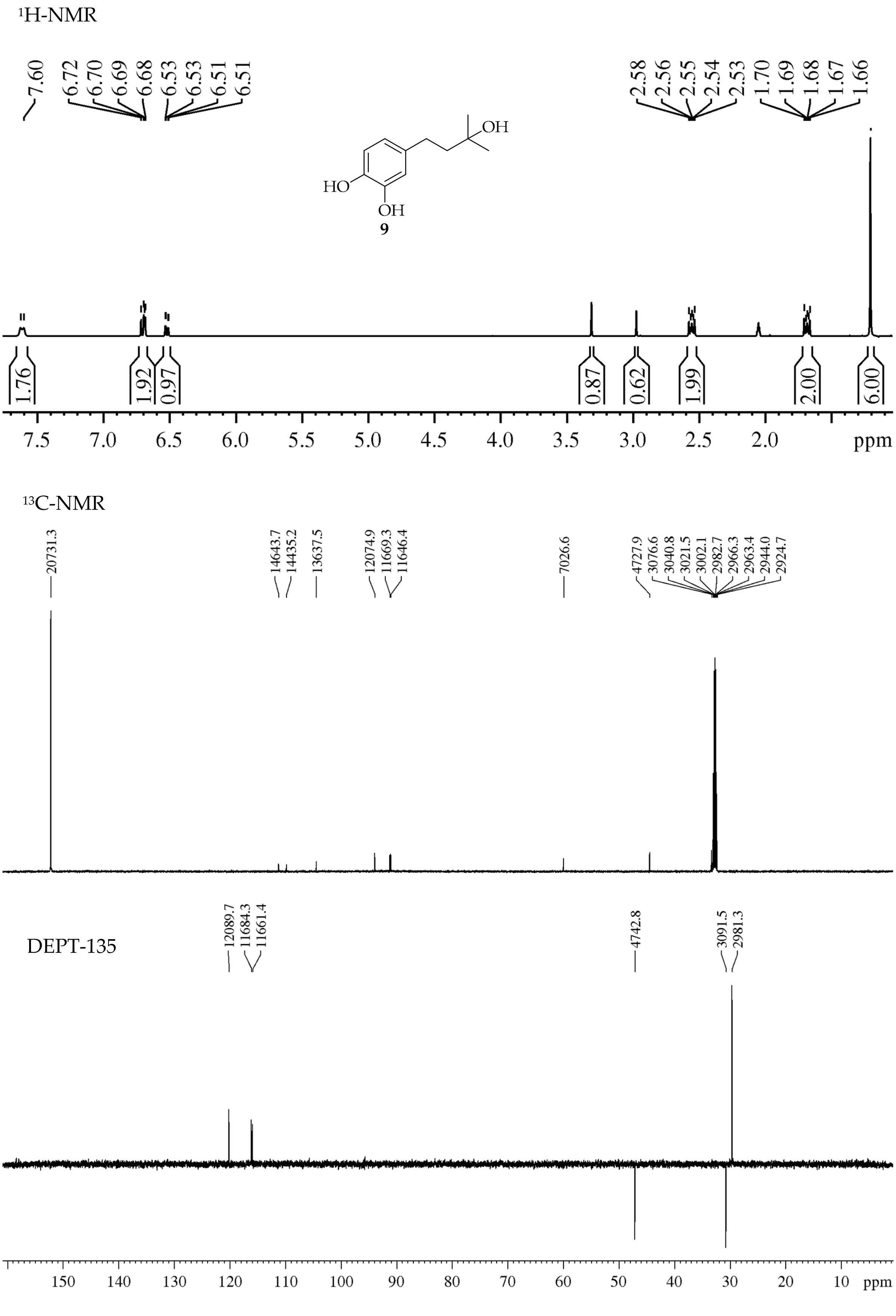

Figure S16: 1D-NMR spectrum of 4-(3-hydroxy-3-methylbutyl)benzene-1,2-diol (**9**).

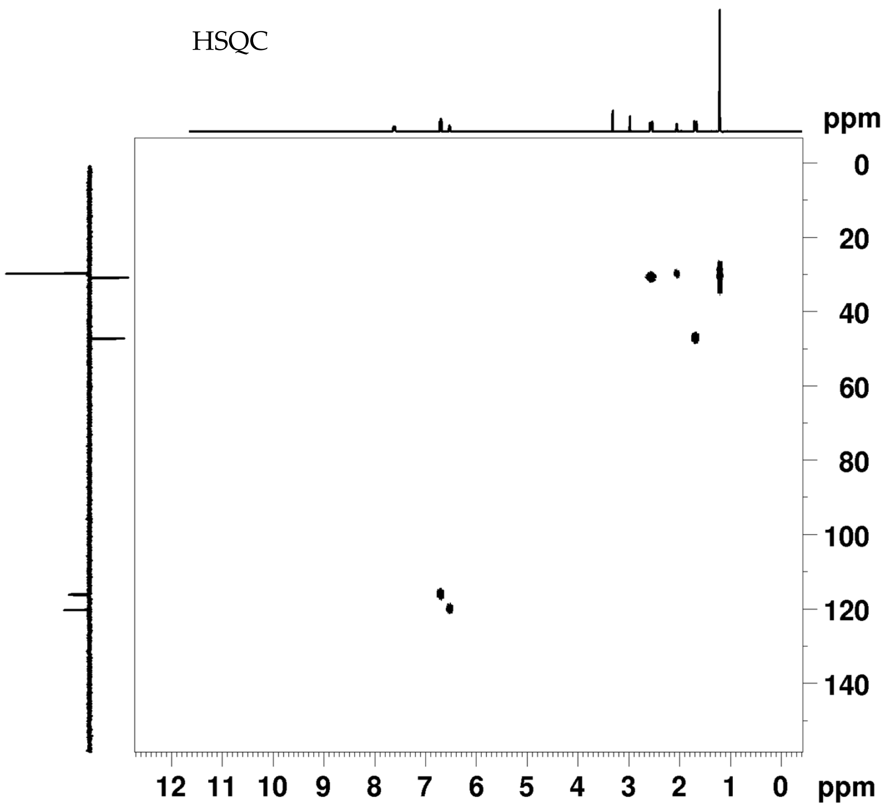

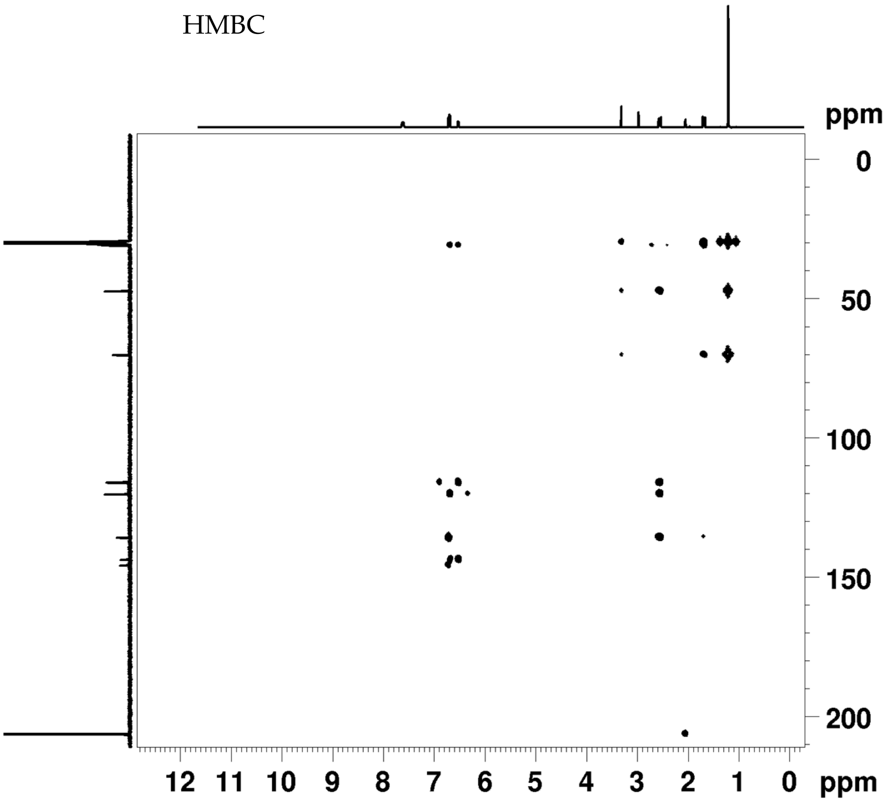

Figure S17: 2D-NMR spectra of 4-(3-hydroxy-3-methylbutyl)benzene-1,2-diol (**9**).

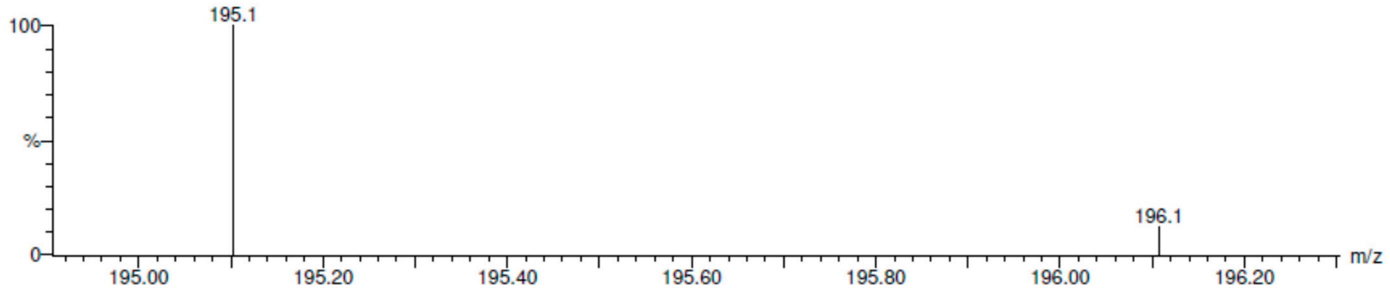

Minimum:	30.00				-1.5			
Maximum:	100.00		5.0	5.0	100.0			
Mass	RA	Calc. Mass	mDa	PPM	DBE	i-FIT	i-FIT (Norm)	Formula
195.1024	100.00	195.1021	0.3	1.5	4.5	22.7	0.0	C11 H15 O3

Figure S18: mass spectrum of 4-(3-hydroxy-3-methylbutyl)benzene-1,2-diol (**9**).
